# Copper homeostasis dysregulation in respiratory diseases: a review of current knowledge

**DOI:** 10.3389/fphys.2024.1243629

**Published:** 2024-05-31

**Authors:** Wei Song, Yuanyi Yue, Qiang Zhang, Xueqing Wang

**Affiliations:** ^1^ Department of Pulmonary and Critical Care Medicine, Shengjing Hospital of China Medical University, Shenyang, China; ^2^ Department of Gastroenterology, Shengjing Hospital of China Medical University, Shenyang, China

**Keywords:** respiratory diseases, immunity, inflammation, copper homeostasis, cuproptosis

## Abstract

Cu is an essential micronutrient for various physiological processes in almost all human cell types. Given the critical role of Cu in a wide range of cellular processes, the local concentrations of Cu and the cellular distribution of Cu transporter proteins in the lung are essential for maintaining a steady-state internal environment. Dysfunctional Cu metabolism or regulatory pathways can lead to an imbalance in Cu homeostasis in the lungs, affecting both acute and chronic pathological processes. Recent studies have identified a new form of Cu-dependent cell death called cuproptosis, which has generated renewed interest in the role of Cu homeostasis in diseases. Cuproptosis differs from other known cell death pathways. This occurs through the direct binding of Cu ions to lipoylated components of the tricarboxylic acid cycle during mitochondrial respiration, leading to the aggregation of lipoylated proteins and the subsequent downregulation of Fe-S cluster proteins, which causes toxic stress to the proteins and ultimately leads to cell death. Here, we discuss the impact of dysregulated Cu homeostasis on the pathogenesis of various respiratory diseases, including asthma, chronic obstructive pulmonary disease, idiopathic interstitial fibrosis, and lung cancer. We also discuss the therapeutic potential of targeting Cu. This study highlights the intricate interplay between copper, cellular processes, and respiratory health. Copper, while essential, must be carefully regulated to maintain the delicate balance between necessity and toxicity in living organisms. This review highlights the need to further investigate the precise mechanisms of copper interactions with infections and immune inflammation in the context of respiratory diseases and explore the potential of therapeutic strategies for copper, cuproptosis, and other related effects.

## Introduction

Cu is a commonly used redox-active metal. Cu ions are involved in a wide range of biochemical reactions by providing or accepting electrons. They bind to various proteins or enzymes as cofactors or structural components in regulating several physiological processes, such as energy metabolism, mitochondrial respiration, inflammatory responses, and antioxidants. At least 30 metalloproteins have been identified as Cu enzymes. Cu enzymes are involved in the oxidation of metals and organic substrates and produce a variety of metabolites, neuropeptides, pigments, and other bioactive compounds (Solomon et al., 2014). In contrast, Cu accumulation can lead to a range of cellular metabolic dysfunctions and, ultimately, cell death (Cobine et al., 2021). Under conventional chemical reactions and physiological conditions, Cu ion uptake and output are tightly regulated in healthy human cells to maintain a dynamic balance of intracellular Cu levels (Ge et al., 2022). An imbalance in Cu homeostasis leads to increased toxicity by inducing different types of cell death, such as apoptosis, paraptosis, pyroptosis, ferroptosis, and macroautophagy/autophagy, thereby inducing the development of various Cu or Cu ion-related diseases (Jian et al., 2020; Guo et al., 2022; Xue et al., 2023).

Recently, a novel cell death pathway triggered by Cu was revealed and named “cuproptosis” by Peter Tsvetkov and colleagues in 2022 (Tsvetkov et al., 2022). Cuproptosis is a form of metal ion-induced regulated cell death. Cu induces apoptosis-independent cell death by directly binding to lipoylated proteins of the tricarboxylic acid (TCA) cycle, inducing aggregation of lipoylated proteins and destabilization of Fe-S cluster proteins, leading to proteotoxic stress. This finding has renewed interest in the application of dysregulated Cu homeostasis in diseases.

Acute and chronic respiratory diseases are significant threats to human health and their incidence is increasing because of deteriorated air quality. In recent years, increasing epidemiological and experimental data have shown significantly elevated Cu levels in the sputum or lung tissues of patients with various chronic lung diseases (Gray et al., 2010; Janssen et al., 2018; Lai et al., 2018). The dysregulation of Cu homeostasis can lead to immune dysregulation, oxidative stress, chronic inflammation, and angiogenic dysfunction, which may contribute to respiratory diseases (Koller et al., 1987; Mandinov et al., 2003; Jiang C. et al., 2021; Chen et al., 2022). Several studies have shown promising results, providing new possible therapeutic targets for this disease. Therefore, this study aimed to summarize the relationship between dysregulated Cu homeostasis and respiratory diseases and explore the potential and effectiveness of clinical treatments.

## Cu homeostasis

Cu is mainly obtained from food, and viscera and shellfish are the richest sources of Cu. Currently, the recommended intake of Cu for adults is 0.8–2.4 mg/day to maintain systemic copper homeostasis. The human body contains 100–200 mg of copper, 50%–70% of which exists in the muscles and bones, 20% in the liver, and 5%–10% in the blood (Bost et al., 2016). With the increase in agricultural, industrial, medical, and metal processing activities in recent years, Cu has been released into ecosystems in large quantities. The continuous increase in Cu content in the environment leads to its increase in water and soil. Therefore, Cu can enter the food chain and accumulate in humans and animals who ingest contaminated food and drinking water (Pastorelli et al., 2012; Tian et al., 2015).

Cu is an important micronutrient necessary for the activity of redox-active enzymes involved in key metabolic reactions, signaling pathways, and biological functions. Transporters and chaperones control Cu ion levels and bioavailability to ensure proper subcellular and systemic distribution of Cu. Dietary Cu is mainly present as Cu^2+^, however, cannot be directly used by cells. Cu uptake occurs mainly through the small intestine, where various reductases exist on the surface of the gastrointestinal (GI) epithelium that reduce Cu^2+^ to Cu^+^ (Miller et al., 2019). The small intestinal epithelium absorbs Cu ions via Cu transport protein 1 (CTR1) or solute carrier family 31 member 1 (SLC31A1). Cu is transported to the other side of the epithelium via the Cu chaperone antioxidant 1 (ATOX1) and ATPase Cu transporter 7A (ATP7A) exported to the bloodstream (Lönnerdal, 2008). Approximately 75% of Cu ions bind to ceruloplasmin (CP) in a non-exchangeable form, and Cu internalization by CTR1 is facilitated by CP, a circulating Cu^2+^-binding protein with Cu-dependent Fe oxidase activity that interacts with the metal reductase STEAP2 on the cell surface, leading to Cu reduction (Maung et al., 2021). Approximately 25% of Cu ions bind to human serum albumin (HSA) in an exchangeable form, and approximately 0.2% of Cu ions bind to histidine for transport to all systems in the body (Cabrera et al., 2008; Kirsipuu et al., 2020). ATOX1 transports Cu to the nucleus, where it binds to transcription factors and drives gene expression. In addition, ATOX1 transfers Cu from the trans-Golgi network (TGN) to Cu-transporting ATPases (ATP7 alpha [ATP7A] and ATP7 beta [ATP7B]). Transporters and chaperones control Cu ion levels and bioavailability to ensure proper subcellular and systemic distribution of Cu. When the Cu content in hepatocytes increases, ATP7B relocates to the bile duct membrane of the hepatocytes and excretes Cu from the biliary tract along with the bile through transmembrane transport. The liver is the leading Cu storage reservoir and the primary organ of the body for Cu excretion. Cu storage function is presumably mediated by metallothionein 1/2 (MT1/2), two thiol-rich proteins that bind Cu ions in a pH-dependent manner via their cysteine residues. The binding of MT1, MT2, and glutathione (GSH) to Cu can limit the cytotoxicity of excess Cu. However, their ability to bind and translocate Cu remains unknown (Hernandez et al., 2008). Plasma proteins, such as CP and albumin, closely regulate Cu under homeostatic conditions and at normal pH. In tissue acidosis, the acidic environment allows weakly bound Cu (II) to be released from exchangeable binding sites on CP or albumin and participates in various biochemical pathways. These include reactive oxygen species (ROS) production, activated protein C inactivation, and endothelial cell proliferation. ATP7A can transfer Cu from the liver to the blood when the peripheral Cu concentration drops, thereby maintaining an effective peripheral circulating Cu concentration. Cu ion absorption, transport, storage, and excretion determine the regulatory processes of Cu metabolic homeostasis. An excess or lack of Cu ions can lead to various diseases.

**FIGURE 1:**
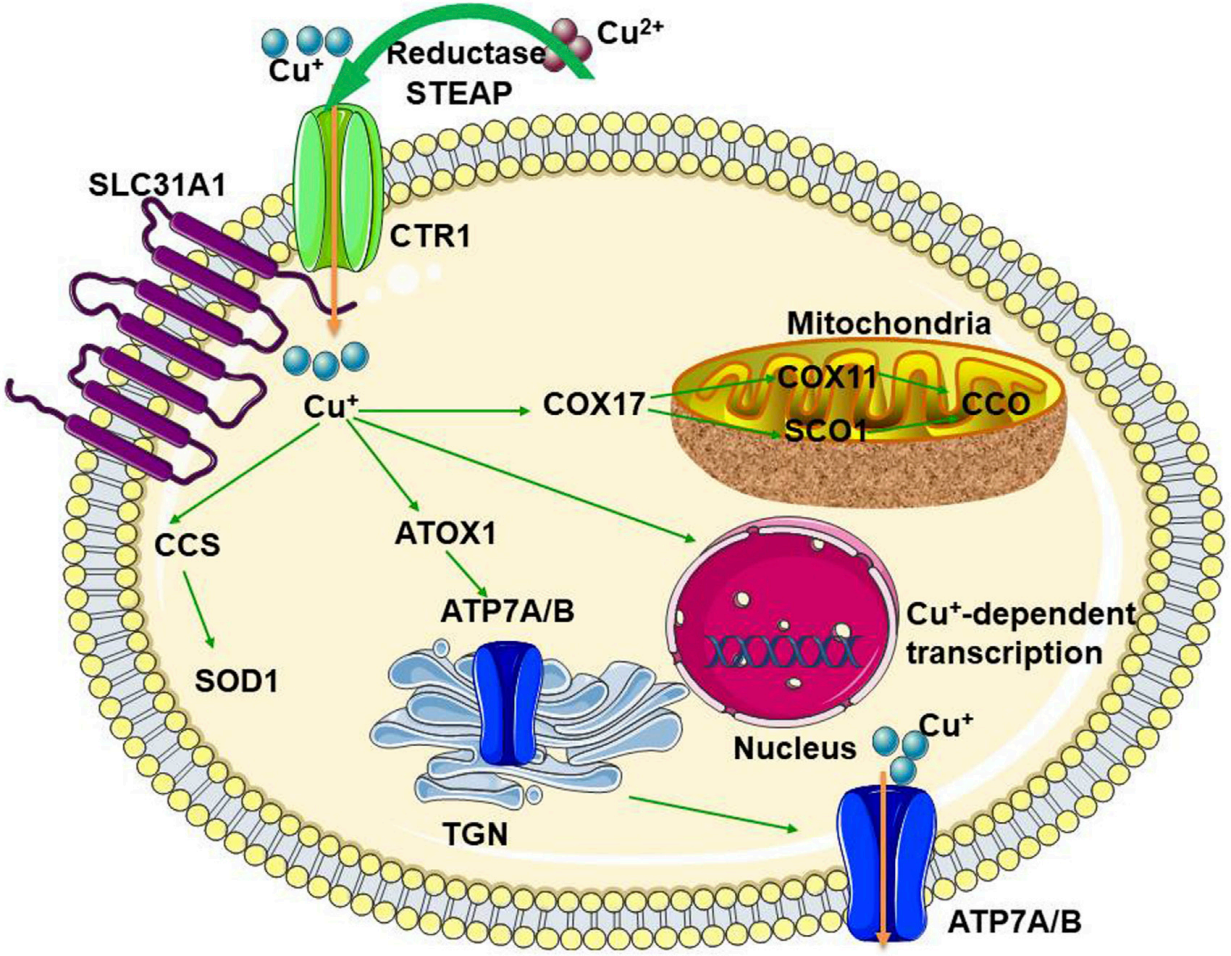
The schematic diagram for general Cu-homeostasis CTR1, Cu transport protein 1; SLC31A1, solute carrier family 31 member 1; CCS, superoxide dismutase; SOD1, superoxide dismutase 1; ATOX1, antioxidant protein 1; ATP7A, ATPase copper transporter 7A; ATP7B, ATPase copper transporting beta; COX, cytochrome c oxidase; SCO1, synthesis of cytochrome oxidase; CCO, cytochrome C oxidase; TGN, trans-Golgi network.

Macrophages, a heterogeneous family of professional phagocytes, are the most abundant immune cells present in the lungs under homeostatic conditions. Macrophage dysfunction leads to chronic lung diseases, including chronic obstructive pulmonary disease (COPD), asthma, and cystic fibrosis (Healy et al., 2021). The Cu importer CTR1 and the ATPase Cu pump ATP7A are induced by interferon (IFN-γ) in macrophages. The ATPase pump is transported to the phagosome chamber and is necessary for the bactericidal activity of the macrophages (White et al., 2009a). IFN-γ-activated macrophages reduce Cu levels in phagosomes to control the fungal pathogen *Histoplamsa capsulatum* (White et al., 2009a). A schematic diagram of general Cu homeostasis is shown in Figure 1.

## Physiological and pathological effects of Cu dysregulation in respiratory diseases

**FIGURE 2:**
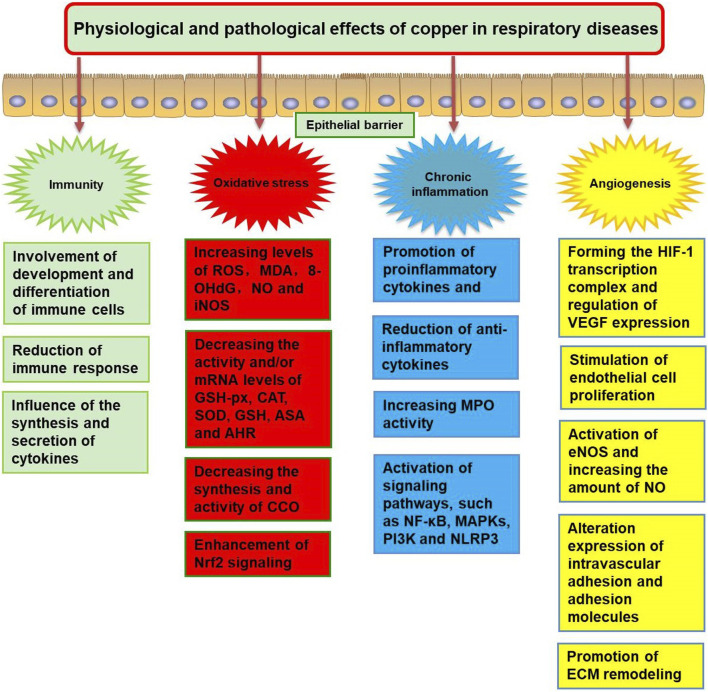
The physiological and pathological effects of copper dysregulation in respiratory diseases. ROS, reactive oxygen species; MDA, malonaldehyde; 8-OHdG, 8-hydroxydeoxyquanosine; NO, nitrogen monoxide; iNOS, inducible nitric oxide synthase; GSH-Px, glutathione peroxidase; CAT, catalase; SOD, superoxide dismutase; ASA, anti-superoxide anion; AHR, anti-hydroxy radical; Nrf2, NFE2 like bZIP transcription factor 2; CCO, cytochrome C oxidase; MPO, myeloperoxidase; NF-κB, nuclear factor-κB; MAPKs, mitogen-activated protein kinases; NLRP3, NOD-like receptor protein 3; PI3K, phosphatidyl-inositol-3-kinase; HIF-1, hypoxia-inducible factor 1; VEGF, vascular endothelial growth factor; eNOS, endothelial nitric oxide synthase; ECM, extracellular matrix.

The physiological and pathological effects of Cu in respiratory diseases include immunity, oxidative stress, chronic inflammation, and angiogenesis. This mechanism is summarized in Figure 2.

### Immunity

Balancing the immune response is vital for lung homeostasis. The pulmonary immune system consists of both the innate and adaptive immune systems, which play an essential role in lung diseases, such as idiopathic interstitial fibrosis (IPF), COPD, and asthma (Feng et al., 2020; Giacalone et al., 2020; Gopallawa et al., 2023; Zhao et al., 2023). Both clinical studies and molecular validation have indicated the critical properties of Cu in regulating host immunity. Cu is involved in the development and differentiation of immune cells. In animal models, Cu deficiency and excess intake reduced several aspects of the immune response, including neutrophil count, lymphocyte proliferation, and production of antigen-specific antibodies (Pocino et al., 1991; Massie et al., 1993; Percival, 1998; Bonham et al., 2002). For example, a study in rats found that antibody titers and natural killer cell cytotoxicity were distinctly repressed after a Cu-deficient diet (Koller et al., 1987). The concentration of monocytes in rat splenic blood decreases due to Cu deficiency (Bala et al., 1991). Cu deficiency reduces interleukin (IL)-2 mRNA expression and protein secretion in activated Jurkat human T cells by inhibiting *IL-2* transcription (Hopkins and Failla, 1999). Cu-deficient mice have smaller thymuses, enlarged spleens, neutropenia, and fewer T cells. Mitogen-induced T-cell proliferation is impaired by the function of B and NK cells (Stabel and Spears, 1989; Cheng et al., 2022). Deficiency or minimal intake of Cu can also impair the number and function of immune cells in the human body (Kelley et al., 1995). Elevated dietary Cu content leads to an unsustainable immune response, including increased production of ROS by macrophages and neutrophils, impaired phagocytosis, neutrophil extracellular trap formation, increased degranulation, and mitochondria-mediated apoptosis (Bala et al., 1991; Monteith and Skaar, 2021). Cu also affects the synthesis and secretion of cytokines that regulate the activity of immune and other cell types (Muñoz et al., 2007). A study of men on a long-term high-Cu diet found that high Cu intake significantly reduced the percentage of circulating neutrophils and serum IL-2R; this implied a decrease in lymphocyte proliferation because IL-2R regulates lymphocyte proliferation, and its concentration decreases with increasing Cu intake (Turnlund et al., 2004). This is consistent with reduced lymphocyte proliferation in aged mice after high Cu treatment against those with naturally obtained serum Cu concentrations (Massie et al., 1993). The results of current human studies support claims based on animal models, in which both Cu deficiency and excess intake modulate immune responses.

### Oxidative stress and antioxidant capacity of Cu

ROS-induced oxidative stress plays a crucial role in the pathogenesis of many diseases, particularly chronic respiratory diseases, such as asthma, COPD, respiratory infections, lung cancer, and interstitial lung disease (Ramírez-Prieto et al., 2006; Park et al., 2009). Metals, such as Cu, have been identified as pro-oxidants and may accelerate the production of free radicals, leading to oxidative stress (Yang et al., 2023). The toxicity of excess Cu is mainly related to the production of reactive oxygen, which mediate the formation of ROS and ROS-induced damage to the organism at all levels (Jomova et al., 2022). Cu ions cycle between the oxidized and reduced states to form hydroxyl radicals. Hydroxyl radicals react with DNA and lipids, causing DNA damage and lipid peroxidation, respectively (Chen et al., 2023). Jiang et al. found that the inflammatory response in patients with various chronic lung diseases promoted the uptake of Cu, which, in turn, caused ROS generation and apoptosis in airway epithelial cells. CuSO_4_ increases ROS production; malonaldehyde (MDA), 8-hydroxydeoxyquanosine (8-OHdG), and nitrogen monoxide (NO) content; inducible nitric oxide synthase (iNOS) activity, and mRNA expression levels in mice (Jian et al., 2020). Simultaneously, CuSO_4_ decreased the activity and mRNA expression levels of antioxidant enzymes, such as glutathione peroxidase (GSH-PX), catalase (CAT), and superoxide dismutase (SOD); decreased the content of GSH; and decreased the antioxidant abilities of anti-superoxide anion (ASA) and anti-hydroxy radical (AHR). CuSO_4_ also induces apoptosis and decreases the mRNA and protein expression of Bcl-2 and Bcl-xL. The mRNA and protein expression levels of Bax, Bak, cleaved-caspase-3, and cleaved-caspase-9 and the Bax/Bcl-2 ratio were increased (Jian et al., 2020). Exposure to CuO nanoparticles (NPs) for up to 24 h in epithelial lung cells (A549) showed very high cytotoxic effects, including lysosomal pathway activation, lysosomal dissolution, significantly increased oxidative damage, and autophagic cell death (Moschini et al., 2013).

Some components of the oxidative defense system, such as SOD, CP, GSH, and metallothionein, are damaged when Cu is deficient. SOD represents the first enzymatic defense system against radical damage by oxygen and possesses three isoforms: cytosolic Cu-Zn SOD1, mitochondrial manganese-dependent SOD2, and extracellular Cu-Zn SOD3 (Uriu-Adams and Keen, 2005). Cu, Zn-superoxide dismutase (Cu, Zn-SOD) is a cytoplasmic enzyme in which Cu and Zn are important components that coordinate its structural and catalytic properties. They are abundant in most tissues, in particular, the bronchial and alveolar epithelial cells, mesenchymal cells, fibroblasts, and endothelial cells (Comhair et al., 2005). A 50%–60% reduction in Cu/Zn SOD activity can lead to severe oxidative stress and cell death (Capriotti et al., 2022). Although Cu is required for ferredoxin function and its absence reduces CP activity, it does not affect the synthesis or secretion of CP, which is synthesized in the liver. Therefore, low CP activity is a common feature of Cu-deficient animals (Prohaska and Brokate, 2002). Metallothioneins are also involved in the homeostatic control of Cu and can bind and activate Cu under reducing conditions (Fabisiak et al., 1999).

Mitochondria coordinate cellular metabolic processes, and micronutrients, such as Cu, are essential for normal mitochondrial effects. In the mitochondria, Cu is an important component of complex IV, also known as cytochrome C oxidase (CCO), which activates enzymes in the respiratory chain (Bomer et al., 2022). Cu deficiency leads to reduced transport of Cu through cytochrome c oxidase (COX)17 for the synthesis of cytochrome oxidase (SCO)1/SCO2 and COX11, which reduces the synthesis of CCO. Cu deficiency decreases CCO activity (Johnson and Newman, 2003). In addition, Cu deficiency can lead to mitochondrial dysfunction by inducing the expression of other mitochondria-associated molecules (Chen et al., 2023).

The main regulator of cellular redox balance is the transcription factor NF-E2-related factor 2 (Nrf2), which inhibits oxidative stress, inflammation, and apoptosis (Han et al., 2023). Cu-induced oxidative stress stimulated Nrf2 recruitment to the peroxisome proliferators-activated receptor γ (PPARγ) promoter, inducing transcription of the target gene and subsequent lipogenesis (Zhong et al., 2022). In addition, Cu exposure increases the nuclear accumulation of Nrf2 in the piscine brain model and increases its ability to bind Cu/Zn SOD. Disulfiram/Cu (DSF/Cu) treatment significantly increased Nrf2 protein expression, nuclear translocation, and transcriptional activity and subsequently upregulated the expression of Nrf2 downstream proteins, including NQO-1 and HO-1. The application of ML385 (an Nrf2 inhibitor) can mitigate this phenomenon. DSF/Cu dramatically activates the phosphorylation of p62, which facilitates competitive binding to Keap1 for degradation, thereby prolonging the half-life of Nrf2 (Jiang et al., 2014). Nrf2 is a potential therapeutic target for lung diseases, such as IPF, asthma, COPD, and acute respiratory distress syndrome (Lee et al., 2021; Albano et al., 2022). In addition, hypoxia-inducible factor 1α (HIF-1α) is a transcription factor that regulates cell response to hypoxia. Cu regulates the target gene selectivity of HIF-1α by at least partially influencing the effect of the binding site of HIF-1α to the target gene (Liu et al., 2018).

### Chronic inflammation

Inflammation is a fundamental mechanism that maintains homeostasis in response to tissue damage (Deng et al., 2023). Persistent respiratory tract inflammation underlies the pathogenesis of many chronic lung diseases, such as COPD, asthma, and pulmonary fibrosis (Racanelli et al., 2018; Feng et al., 2021a; Zhang et al., 2021). Exposure to high concentrations of Cu is associated with pulmonary inflammation and chronic respiratory disease. For example, Chen et al. reported that Cu exposure promoted the release of the pro-inflammatory cytokines IL-6 and IL-8 in 16HBE cells (Chen et al., 2022). Intratracheal instillation of BeCu alloys in mice results in acute pulmonary lesions, including acute alveolitis and interstitial inflammation. In addition, type II epithelial cell hyperplasia and centriacinar fibrosis were observed 7 days after administration (Benson et al., 2000). In mice, CuSO_4_ causes inflammation by increasing myeloperoxidase (MPO) activity, activating the nuclear factor-κ-B (NF-κB) signaling pathway, and downregulating the mRNA and protein expression levels of anti-inflammatory cytokines (IL-2, IL-4, and IL-10) (Jian et al., 2020). Cu activates many other signaling pathways involved in inflammation, reduces lipid synthesis, and alters cell carbohydrate requirements (Bharathi Devi et al., 2016). Excessive Cu exposure can regulate a large number of cytokines in both directions, increasing and/or decreasing them through a variety of molecular and cellular signaling pathways, including the NF-κB pathway, mitogen-activated protein kinase (MAPKs) pathway, Janus kinase signal transducer and activator of transcription (JAK-STAT) pathway, and NOD-like receptor protein 3 (NLRP3) inflammatory vesicles (Deng et al., 2023). Cu also activates phosphatidyl-inositol-3-kinase (PI3K), an enzyme that activates auto-inflammatory mediators, recruits inflammatory cells and airway remodeling (Reddel et al., 2022), and increases expression of various ILs (e.g., IL-1β, IL-6, IL-8, IL-19) (Winter et al., 2021), iNOS, and pro-inflammatory factors, such as cyclooxygenase-2 (COX-2), thereby inducing chronic diseases, such as metabolic disorders and cancer (Pereira et al., 2016). Elevated Cu levels may be due to the release of Cu from damaged tissues caused by inflammation (Guo et al., 2011a).

Cu deficiency also causes chronic inflammation (Guo et al., 2011b; Zajac, 2021). Liu et al. found reduced Cu levels in chronic TNF-α-dependent lung inflammation and TNF-α overexpressing mice (Liu et al., 2016). This suggests a role for Cu ions in inflammation-induced lung injury and a link between TNF-α and Cu levels, implying that restoring lung Cu status is a potential strategy for treating and preventing chronic lung inflammation and related diseases. Thus, Cu ions may be secondary messengers for inflammation transmission and the inflammatory load response (Winter et al., 2021). However, the regulation of cellular metabolism by Cu requires further investigation.

### Angiogenesis

Angiogenesis is a tightly controlled physiological process that occurs during regeneration, development, and trauma repair. Angiogenesis in the lungs can be stimulated by factors such as hypoxia, extensive lung tissue injury, and oncogene and cytokine activation (Siafakas et al., 2007). After extensive lung injury, inflammatory exudations impede gas exchange and release large amounts of pro-angiogenic factors that promote angiogenesis (Ingbar, 2000). Inflammatory cell activation can also cause relative local oxygen deficiency, triggering hypoxia-induced factors to promote the release of proangiogenic factors and causing compensatory capillary proliferation to adapt to tissue metabolic demands (Pugh and Ratcliffe, 2003). Angiogenesis and inflammatory responses are often associated with various physiological and pathological conditions (Costa et al., 2007). Angiogenesis has been implicated in the development of many lung diseases (Tuder and Voelkel, 2002; Siafakas et al., 2007; Hanumegowda et al., 2012; Eldridge and Wagner, 2019). For example, angiogenesis promotes tumor proliferation and dissemination in lung cancer; hypoxia in COPD leads to the thickening of the vascular wall and regeneration of the pulmonary microvasculature to compensate for physiological requirements; angiogenesis and remodeling promote arterial hypertension in pulmonary hypertension; and capillary hyperplasia and increased permeability in acute lung injury lead to diffuse alveolar edema and hyaline membrane formation.

Endothelial cells play a significant role in angiogenesis, as they are the main targets of molecular signals for growth, proliferation, and migration. There is increasing evidence that Cu plays a significant role in controlling normal endothelial cell growth and proliferation in wound healing and cancer (Mandinov et al., 2003; Lowndes and Harris, 2005). Cu stimulates endothelial cell proliferation under various benign and malignant conditions (Hu, 1998; Li et al., 2012). The mechanism by which Cu regulates endothelial cell growth has not been elucidated but may involve the functions of many Cu-dependent enzymes and transcription factors, including SOD-1, LOX-1, and HIF-1α (Lowndes and Harris, 2005). Cu ions activate endothelial nitric oxide synthase (eNOS) in rat pulmonary artery endothelial cells and increase NO levels by reducing oxidative clearance (Demura et al., 1998). Cu deficiency alters leukocyte expression of intravascular adhesion and adhesion molecules (e.g., ICAM-1/VCAM-1) that activate endothelial cells (ECs) (Schuschke et al., 2001; Schuschke et al., 2002). Chen *et al.* suggested that the Cu chaperone function of ATOX1 was involved in vascular endothelial growth factor (VEGF)-induced angiogenesis by activating lysyl oxidase (LOX) via the CTR1-ATOX1-ATP7A pathway in ECs (Chen et al., 2015). The anti-angiogenic effect of tetrathiomolybdate (TM) also promotes its use in cancer (Brewer, 2003). The ATP7A Cu transporter actively maintains growth factor-dependent angiogenic remodeling by promoting extracellular matrix (ECM) remodeling, CuO protease secretion, and Rac signaling. During these processes, the ATP7A pump and CTR1 provide sufficient Cu to vascular cells (Urso and Maffia, 2015). Bogaard et al. (2012) found that endothelial proliferation and severe pulmonary hypertension occurred only when adequate amounts of dietary Cu were provided to a SuHx rat model. Long-term treatment with TM reversed pulmonary hypertension and vascular occlusion, and TM inhibited the proliferation of cultured human lung endothelial cells from patients with pulmonary arterial hypertension (PAH) and control subjects. In the pulmonary blood vessels of SuHx rats, the therapeutic effect of TM is associated with decreased expression of survivin, increased production of the pro-apoptotic sphingoid metabolite ceramide, and increased expression of apoptosis-inducing factor (AIF) and Bim, a member of the apoptosis-promoting B-cell leukemia/lymphoma (BCL-2 protein family) (Bogaard et al., 2012).

The intersection between Cu metabolism and angiogenic pathways provides a basis for investigating the efficacy of anti-angiogenic therapeutic regimens to reduce Cu levels in the body, thereby slowing the progression of angiogenic disease. Considerable work is needed to obtain more information on the involvement of the Cu transport system in the role of angiogenesis at all stages of angiogenesis.

## Cuproptosis

**FIGURE 3:**
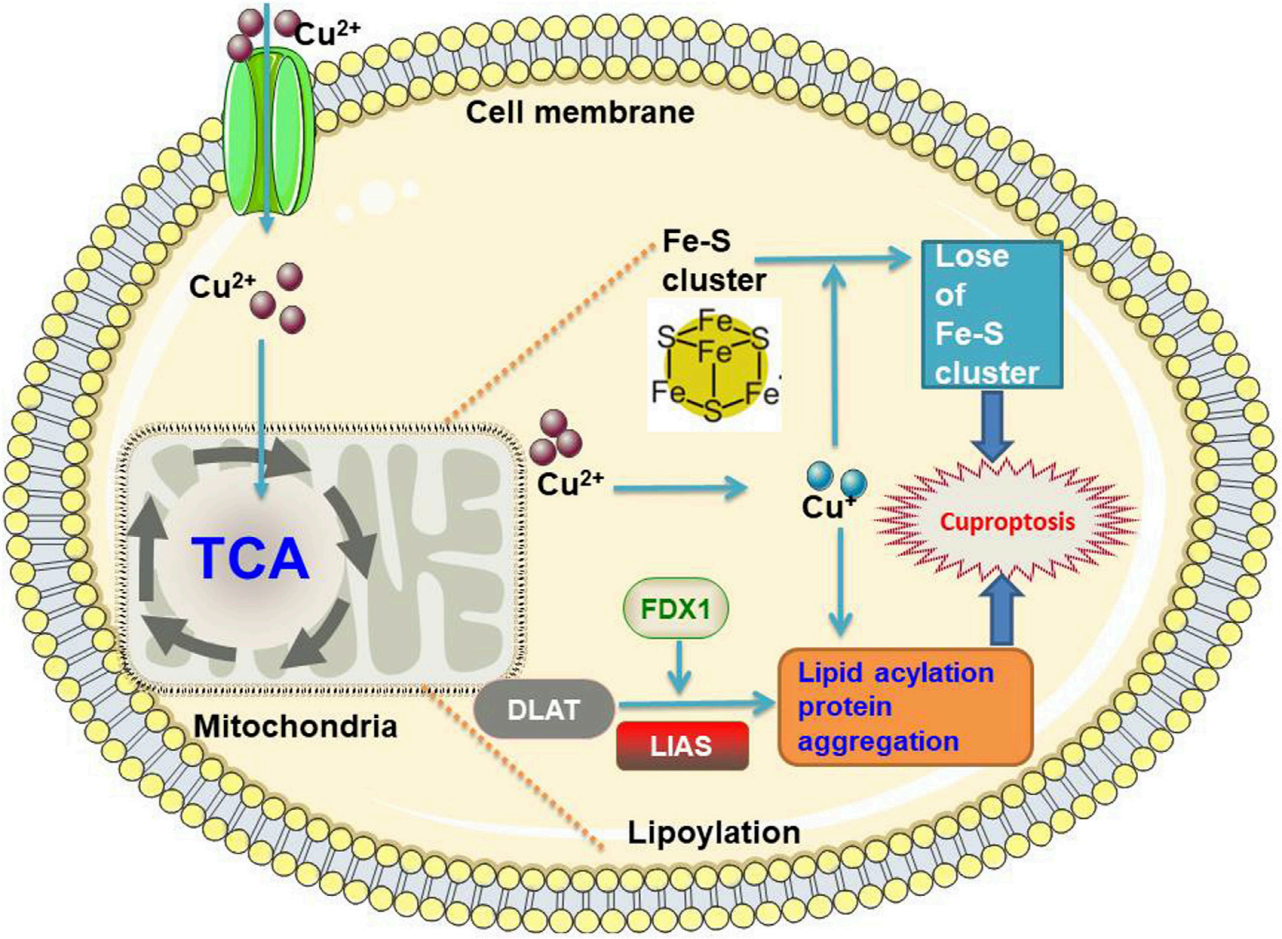
The schematic diagram of cuproptosis FDX1, ferredoxin 1; DLAT, dihydrolipoamide S-acetyltransferase; LIAS, lipoic Acid synthetase; TCA, tricarboxylic acid.

Although cuproptosis was first proposed in 2022, the research on cuproptosis began in 2019. Golub *et al.* identified two small molecules that could carry Cu ions across the cell membrane, disulfiram and elesclomol. These two Cu^2+^ carriers were shown to kill specific drug-resistant cancer cells (Tsvetkov et al., 2019). Tsvetkov et al. referred to this unique form of Cu-dependent cell death as cuproptosis and further proposed a mechanism for elesclomol-induced cell death. Excess intracellular Cu^2+^ can be transported to the mitochondria via ion carriers, where ferredoxin 1 (FDX1) reduces Cu^2+^ to Cu^1+^. In the mitochondria, there is an increase in Cu^+^, which binds directly to lipid-acylated components of the TCA cycle (e.g., dihydrolipoamide S-acetyltransferase (DLAT)), leading to lipid-acylated protein aggregation and destabilization of Fe-S cluster proteins; these result in proteotoxic stress and ultimately cell death, termed cuproptosis. A schematic diagram of cuproptosis is summarized in Figure 3. Notably, the Cu ion carrier-induced cell death pathway cannot be treated with inhibitors of other known cell death pathways, such as pan-cysteine aspartase inhibitors (anti-apoptotic compounds), Fe inhibitor-1 (an anti-Fe apoptotic compound), necrosin-1 (an anti-apoptotic compound), and N-acetylcysteine (an oxidative stress inhibitor), suggesting that cuproptosis mechanisms are distinct from previously identified cell death pathways, *e.g.*, apoptosis, necroptosis, pyroptosis, and ferroptosis (Cui et al., 2022; Tong et al., 2022). Cuproposin plays a key role in tumor cell proliferation, metastasis, and drug resistance (Yu et al., 2020; Tong et al., 2022). The link between Cu and hypoxia is complex. Similar to mitochondrial diseases, the clinical phenotype of Fe-S disease is associated with respiratory defects and severe metabolic dysfunction (Lill and Freibert, 2020; Braymer et al., 2021). Hypoxic conditions inhibit antioxidant defense mechanisms by increasing ROS levels, Cu transport, and mitotic phagocytosis, thereby promoting Cu cytotoxicity and inhibiting cuproptosis, leading to abnormal cell survival and proliferation. Specific molecular mechanisms may involve the metal regulatory transcription factor 1 (MTF1) and forkhead box O-3 (FoxO3) signaling pathways (Pan et al., 2020; Zhao et al., 2022). There are 12 genes involved in the cuproptosis pathway, including seven pro-cuproptosis genes (FDX1, lipoic acid synthetase (LIAS), lipoyltransferase 1 (LIPT1), dihydrolipoamide dehydrogenase (DLD), DLAT, pyruvate dehydrogenase E1 subunit alpha 1 (PDHA1), and pyruvate dehydrogenase E1 subunit beta (PDHB)), three anti-cuproptosis genes (MTF1, glutaminase (GLS), and cyclin-dependent kinase inhibitor 2A (CDKN2A)), and two Cu transporters (ATP7B and SLC31A1) (Liu and Tang, 2022a).

FDX1 is a key regulator of Cu ion carrier-induced cell death (Tsvetkov et al., 2022). It promotes ATP production and participates in the metabolism of glucose, fatty acids, and amino acids in lung adenocarcinomas (Zhang et al., 2021). FDX1 targets six important components of the lipoic acid pathway: LIPT1, LIAS, DLD, DLAT, PDHA1, and PHDB. It is also a key mediator of protein-lipid acylation, making it an important promoter of cellular processes and regulator of cuproptosis (Tsvetkov et al., 2022). Increased FDX1 activity and mitochondria-dependent energy metabolism are strong inducers of the cytotoxic effects of elesclomol-mediated cuproptosis (Tsvetkov et al., 2019) and inhibit Fe-S cluster biosynthesis (Tsvetkov et al., 2019). In addition, cuproptosis caused by depletion of the natural intracellular Cu chaperone GSH is associated with reduced lipid acylation and increased DLAT oligomerization (Tsvetkov et al., 2022). Yun et al. identified FDX1-rs10488764 as a lung cancer risk polymorphism among smokers and non-smokers as well as three different pathological subgroups, which provided new clues to the role of FDX1 in disease development (Yun et al., 2022). However, limited information is available regarding the association between FDX1 and lung cancer. ATP7B is an essential Cu transporter that regulates Cu transport from the cytoplasm to the Golgi apparatus and lysosomes to maintain Cu homeostasis (Polishchuk et al., 2014). Nonetheless, high Cu levels alter the localization of ATP7B to lysosomes, leading to the release of Cu via vesicular transport (Bartee and Lutsenko, 2007). Yu et al. identified a novel regQTL-SNP, rs3768617, which may have an impact on lung cancer risk by affecting the expression of microRNA (miR)-548b-3p and laminin subunit gamma 1 (LAMC1) (Yu et al., 2021). Therefore, in addition to genes, it is possible to explore the role of SNPs in cuproptosis-associated lncRNAs, miRNAs, and lung cancer risk and identify novel regQTL-SNPs associated with lung cancer risk in future studies. The Cu-associated genes are listed in Table 1. These genes included *CP* (Matsuoka et al., 2018), Cu transporter 2 (*CTR2*) (Chang et al., 2022), *ATOX1* (Chen et al., 2015), Cu chaperone for superoxide dismutase (*CCS*) (Hu et al., 2023), Cu chaperone for cytochrome c oxidase (*COX*) *11* (Ge et al., 2022), *COX17* (Suzuki et al., 2003), synthesis of cytochrome oxidase (*SCO*) *1* (Zhang et al., 2021), *SCO2* (Liu et al., 2018), cytochrome c oxidase assembly factor 6 (*COA6*) (Zhang et al., 2023), solute carrier family 25 member 3 (*SLC25A3*), *MT1* and *MT2* (Eckschlager et al., 2009; Sauler et al., 2022), subunits 1 and 2 of cytochrome c oxidase (*COX1* and *MT-CO2*), *SOD1* (Skórska et al., 2021; Li et al., 2022), tyrosinase (*TYR*), lysyl oxidase like 2 (*LOXL2*) (Aumiller et al., 2017; Peng et al., 2017), dopamine β-hydroxylase (*DBH*), amine oxidase 3 (*AOC3*) (Ovet and Oztay, 2014; Chang et al., 2021), Mitogen-activated protein kinase kinase 1/2 (*MEK1*/*MEK2*), unc51-like kinase 1/2 (*ULK1*/*ULK2*) (Cheng et al., 2019), pyruvate dehydrogenase kinase 1 (*PDK1*) (Liu and Yin, 2017; Zhang et al., 2023), phosphodiesterase 3B (*PDE3B*), conjugating enzyme E2 (*UBE2*)*D1*, *UBE2D2*, *UBE2D3, UBE2D4*, histone H3.1 and histone H4 in the H3/H4 tetramer (*H3C1* and *HC14*), vascular endothelial growth factor A (*VEGFA*) (Zhang et al., 2015), programmed cell death 1 ligand 1 (*PD1L1*) (Qiu et al., 2019), *FDX1* (Zhang et al., 2021), *LIAS* (Cai et al., 2022), *DLD*, *DLAT* (Liu and Tang, 2022b), *PDHA1* (Deng et al., 2022), *PDHB*, *MTF1*, *GLS* (Liu and Tang, 2022b), *CDKN2A* (Peng et al., 2022), *SLC31A1* (Liu and Tang, 2022b), *ATP7A,* and *ATP7B* (Liu and Tang, 2022b).

**TABLE 1:** Cu-associated genes.

Gene	Name	Clinical values	References
*CP*	Ceruloplasmin	Major exchangeable plasma Cu carrier; CP is produced heterotopically in lung adenocarcinoma cells and its expression is associated with tumor progression	Matsuoka et al. (2018)
*CTR2* (also known as *SLC31A2*)	Copper transporter 2	CTR1 regulator; The CMRGS composed of 3 key genes (LOXL2, SLC31A2 and SOD3) had showed good clinical value and stratification ability in the prognostic assessment of LUAD	Chang et al. (2022)
*ATOX1*	Antioxidant protein 1	Cytosolic Cu metallochaperone; It plays a role in pneumonia neovascularization	Chen et al. (2015)
*CCS*	Copper chaperone for superoxide dismutase	Cytosolic Cu metallochaperone; LUAD with higher CCS levels has poorer prognosis	Hu et al. (2023)
*COX11*	Copper chaperone for cytochrome c oxidase 11	Mitochondrial Cu metallochaperone	Ge et al. (2022)
*COX17*	Copper chaperone for cytochrome c oxidase 17	Mitochondrial Cu metallochaperone; COX17 is upregulated in NSCLC	Suzuki et al. (2003)
*SCO1*	Synthesis of cytochrome oxidase 1	Mitochondrial Cu metallochaperone; Associated with poor prognosis in LUAD	Zhang et al. (2021c)
*SCO2*	Synthesis of cytochrome oxidase 2	Mitochondrial Cu metallochaperone; High expression is associated with poor prognosis of LUAD	Liu et al. (2018b)
*COA6*	Cytochrome c oxidase assembly factor 6	Mitochondrial Cu metallochaperone; High expression is related to unfavorable prognosis and enhanced oxidative phosphorylation in LUAD.	Zhang et al. (2023a)
*SLC25A3*	Solute carrier family 25 member 3	Mitochondrial Cu importer	
*MT1* and *MT2*	Metallothionein	Cu/Zn storage protein; Macrophage subsets with high metallothionein expression were found in advanced COPD; Prognostic factors for progression and drug resistance in NSCLC	Eckschlager et al. (2009), Sauler et al. (2022)
*COX1* and *MT-CO2*	Subunits 1 and 2 of cytochrome c oxidase	Respiratory O_2_ reduction	
*SOD1*	Superoxide dismutase 1	Superoxide scavenger; SOD1 gene expression is increased in COPD patients; Increased serum SOD1 concentration increases all-cause mortality in lung cancer patients	Skórska et al. (2021), Li et al. (2022a)
*TYR*	Tyrosinase	Tyrosine oxidation	
*LOXL2*	Lysyl oxidase like-protein 2	Lysine oxidation; A critical role in ECM remodeling; Positive correlation between LOXL2 serum levels and disease severity and progression in IPF patients; LOXL2 plays an important role in lung cancer progression and metastasis	Aumiller et al. (2017), Peng et al. (2017)
*DBH*	Dopamine β-hydroxylase	Dopamine oxidation	
*AOC3* (also known as *VAP1*)	Amine oxidase 3 (vascular adhesion protein 1)	Amine oxidation; Low expression of AOC3 leads to poor clinical outcome and lymph node metastasis of lung cancer; Promote the development of pulmonary fibrosis	Ovet and Oztay (2014), Chang et al. (2021)
*MEK1* and *MEK2* (also known as *MAP2K1* and *MAPK2*)	Mitogen-activated protein kinase kinase 1/2	Protein kinase	
*ULK1/ULK2*	Unc51-like kinase 1/2	Protein kinase; Ulk2 overexpression inhibits proliferation and enhances cisplatin sensitivity in NSCLC	Cheng et al. (2019)
*PDK1*	Pyruvate dehydrogenase kinase 1	Protein kinase; PDK1 is involved in pulmonary vascular remodeling, leading to hypoxic pulmonary hypertension; PDK1 expression is significantly upregulated in NSCLC tissues and correlates with advanced T-stage	Liu and Yin (2017), Zhang et al. (2023b)
*PDE3B*	Phosphodiesterase 3B	Cyclic AMP degradation	
*UBE2D1*, *UBE2D2*, *UBE2D3* and *UBE2D4*	E2D ubiquitin Conjugating enzyme E2 D1, D2, D3 and D4	Ubiquitin conjugation to protein target	
*H3C1* and *HC14*	Histone H3.1 and Histone H4 in the H3/H4 tetramer	Copper reductase	
*VEGFA*	Vascular endothelial growth factor A	Growth factor; VEGFA has been identified as a major regulator of tumor angiogenesis and patients with high levels of VEGFA mRNA expression have a shorter survival time	Zhang et al. (2015)
*PD1L1* (also known as *CD274*)	Programmed cell death 1 ligand 1	Immune response control; Immunotherapy of the PD-1/L1 axis provides long-term survival benefits for patients with wild-type advanced NSCLC with acceptable adverse reactions	Qiu et al. (2019)
*FDX1*	Ferredoxin 1	CRG; Upregulated in LUAD and LUSC; FDX1 high expression correlated with better prognosis in many cancers	Zhang et al. (2021b)
*LIAS*	Lipoic Acid Synthetase	CRG; Upregulated in LUAD and LUSC; LIAS high expression led to worse OS and FP, and correlated with more advanced lung cancer staging	Cai et al. (2022)
*DLD*	Dihydrolipoamide Dehydrogenase	CRG	
*DLAT*	Dihydrolipoamide S-Acetyltransferase	CRG; Upregulated in LUAD and LUSC.	Liu and Tang (2022b)
*PDHA1*	Pyruvate Dehydrogenase E1 Subunit Alpha 1	CRG; LUAD patients with high PDHA1 levels are significantly associated with poor OS and FP outcomes	Deng et al. (2022)
*PDHB*	Pyruvate Dehydrogenase E1 Subunit Beta	CRG	
*MTF1*	Metal Regulatory Transcription Factor 1	CRG	
*GLS*	Glutaminase	CRG; high expression led to worse OS	Liu and Tang (2022b)
*CDKN2A*	Cyclin Dependent Kinase Inhibitor 2A	CRG; CDKN2A higher expression led to risk in LUAD	Peng et al. (2022)
*SLC31A1*	Solute Carrier Family 31 Member 1	CRG; SLC31A1 alleles were associated with a worse prognosis; Upregulated in LUAD and LUSC.	Liu and Tang (2022b)
*ATP7A*	ATPase Copper Transporting Alpha	CRG	
*ATP7B*	ATPase Copper Transporting Beta	CRG; ATP7B alleles were associated with a decreased risk of LUAD	Liu and Tang (2022b)

CRG, cuproptosis-related gene; LUAD, lung adenocarcinoma; LUSC, lung squamous cell; ECM, extracellular matrix; NSCLC, non-small cell lung cancer; COPD, chronic obstructive pulmonary disease; IPF, idiopathic pulmonary fibrosis; OS, overall survival; FP, first progression.

## Dysregulation of Cu homeostasis in respiratory diseases

### Lung cancer

Lung cancer is one of the most common malignant cancers, accounting for 11.6% of all diagnoses and 18.4% of deaths associated with the disease worldwide (Arffman et al., 2019; Huang et al., 2020). Early detection, diagnosis, and treatment are key to reducing mortality and improving disease prognosis (Terra et al., 2021). High serum Cu concentrations and the Cu:Zn ratio have been reported to be associated with lung cancer (Wang et al., 2021). Zhang et al. conducted a meta-analysis of 33 studies (including 3026 lung cancer cases) and suggested that an increase in circulating serum Cu concentration was associated with an increased risk of lung cancer (Zhang and Yang, 2018). A recent meta-analysis of 39 observational studies showed that serum Cu-to-Zn ratios were significantly higher in patients with lung cancer than in healthy controls and patients with benign lung disease (Zhang et al., 2022). Studies have demonstrated the role of cuproptosis in lung cancer. For example, Wang *et al.* (Wang et al., 2023) found that ATP7A and ATP7B harbored the highest mutation frequency in lung adenocarcinoma (LUAD), followed by DLD, PDHA1, DBT, DLST, DLAT, and FDX1. Based on the 13 recognized CRGs, patients with LUAD could be well differentiated into two different molecular subgroups, and the prognostic model based on the CRGs-risk score presented a robust and effective predictive ability; this served as an independent prognostic indicator for patients with LUAD. In addition, Liu et al. (2022) established a cuproptosis score to quantify its related pattern of individual patients with LUAD and found that the cuproptosis score was a potential independent prognostic factor of LUAD in these cluster cases. Moreover, Luo et al. (2023) established a prognostic cuproptosis-related immune gene (CRIMGs) score and demonstrated that this score could serve as a promising indicator of prognosis and immunotherapeutic response in patients with non-small-cell lung cancer (NSCLC). Zheng et al. (2022) identified 34 prognostic cuproptosis-related lncRNAs (CR-lncRNAs) in two new molecular subtypes and constructed a novel prognostic model for LUAD. Furthermore, CR-lncRNAs are associated with changes in immune cell infiltration in LUAD (Wang et al., 2022).

However, the underlying mechanism of action of Cu in lung cancer remains unclear. Recent studies have shown that Cu can activate HIF-1α-related pathways to stimulate the production of VEGF (Lelièvre et al., 2020), leading to tumor angiogenesis, including lung cancer (Zimna and Kurpisz, 2015; Wu et al., 2019). Cu has also been reported to activate agammaglobulinemia tyrosine kinases (ATK). Moreover, Cu directly or indirectly activates the PI3K/AKT pathway (Ostrakhovitch et al., 2002; He et al., 2019). Furthermore, Cu-related enzymes are associated with lung metastases in other cancers. For example, a mediator of cell motility 1 (MEMO1) is a Memo, a Cu-dependent redox protein identified as an intracellular Cu-dependent protein essential for migration and invasion of breast cancer cells *in vitro* and spontaneous lung metastasis *in vivo* (MacDonald et al., 2014). Identifying appropriate methods for blocking Cu I) binding sites on MEMO1 may be a potential approach for releasing Cu ions and inhibiting tumor cell metastasis (Tong et al., 2022). Therefore, elucidating the mechanism of cuproptosis and targeting cell Cu death pathways is crucial for developing future combination therapies. However, systematic model biology and clinical studies on the application of Cu in lung cancer are lacking. Future clinical trials using biomarker-driven approaches with Cu ion carriers should be conducted to further explore the treatment of lung cancer sensitive to cuproptosis (Tsvetkov et al., 2022).

### COPD

COPD is a heterogeneous lung disease characterized by chronic respiratory symptoms caused by bronchitis, bronchiolitis, and/or emphysema. Oxidative stress and chronic inflammation are important features of the pathogenesis of COPD (Zhang et al., 2020; Feng et al., 2021a; Feng et al., 2021b; Zhang et al., 2022; Zhao et al., 2022; Agustí et al., 2023). Cross-sectional data from the 2015–2016 National Health and Nutrition Examination Survey (NHANES) suggest that high levels of Cu may increase the risk of COPD in men of the United States, with higher Cu concentrations in the COPD group (124.30 μg/dL) than in the healthy group (114.25 μg/dL); however, neither group exceeded the normal range (63.5–158.9 μg/dL) (Fei et al., 2022). Jiang et al. found that the level of sputum Cu increased in patients with various pulmonary diseases, especially COPD and asthma (Jiang et al., 2021). Cu levels are strongly negatively correlated with lung function, with high serum Cu levels associated with low forced expiratory volume in 1 s (FEV1) (Pearson et al., 2005). There were no significant differences in Zn and Cu/Zn ratios between COPD smokers and non-smokers, but there were differences in Cu, MDA, and serum paraoxonase 1 (PON1) activities (Sarioglu et al., 2020). However, COPD exacerbations have significantly higher levels of Cu and Zn but lower Cu/Zn ratios than those in the control group (Bozkus et al., 2016). The exhaled breath condensate (EBC) of patients with stable COPD had lower Cu levels than those of the controls, and Cu levels in the EBC were positively related to FEV1 in subjects with COPD (Mutti et al., 2006). Elevated Cu levels have also been found in the lungs of rats with COPD (Qi et al., 2023). Cu deficiency induces emphysema in animal models (Mizuno et al., 2012). Therefore, appropriate Cu levels are essential for proper respiratory function (Rucker et al., 1998). The increase in systemic Cu cannot be explained by nutritional intake and may be associated with inflammatory phenomena and/or susceptibility to tobacco (Dransfield et al., 2006; Hakim et al., 2012; Maury et al., 2015).

Loss of Cu directly affects the integrity of alveolar epithelial cells because elastin synthesis is regulated through the Cu-dependent enzyme lysine oxidase (Janssen et al., 2018). An imbalance in elastase-elastase inhibitors is a major cause of emphysema development (Hisata et al., 2021). Animals fed a Cu-deficient diet also develop emphysema, as Cu deficiency prevents LOX activation (Zajac, 2021). LOXs are Cu-dependent enzymes, including LOX, LOXL1, LOXL2, LOXL3, and LOXL4, whose main function is to promote the covalent crosslinking of ECM proteins, such as collagen and elastin. LOX is important for establishing the structural integrity and stability of the ECM in individual organs and is the most important enzyme for ECM crosslinking. LOX becomes active when bound to Cu in the Golgi apparatus and is secreted extracellularly to crosslink elastin and collagen. Cu can activate lung fibroblasts through the TGF-β/Smad pathway that is activated by LOXL2, leading to pulmonary fibrosis and, further, COPD (Fei et al., 2022). Lox-deficient mice show abnormal bronchial formation with thick airway walls (Mäki et al., 2005). Thus, intracellular Cu levels determine LOX activity. LOX plays a decisive role in the healing of lung parenchyma after injury (Janssen et al., 2019). Menkes’ disease is a congenital Cu deficiency disorder that leads to emphysema (Mizuno et al., 2012). Moreover, elastin abnormalities may be caused by a deficiency in the Cu-dependent enzyme LOX (Grange et al., 2005). COPD is also associated with cigarette smoking and exposure to other environmental factors. Kankanamage found that nitrosamine metabolites produced by smoking and e-cigarettes can promote high rates of DNA oxidation in the presence of Cu^2+^ and that NADPH causes DNA oxidation (Kankanamage et al., 2020). The levels of antioxidant parameters, such as SOD, catalase, GPx, GSH, and GSH peroxidase (GR), decreased with increasing disease severity. Antioxidant markers are inversely correlated with the severity of COPD (Singh et al., 2017); however, the specific pathways involving Cu ions require further investigation.

Qi et al. found that 18 key Cu metabolization-related genes were significantly enriched in signaling pathways and biological processes associated with COPD development. DLD and CDKN2A are abnormally expressed in the airway epithelial cells of patients with COPD, and smoking can promote their expression to a certain extent, which has been verified in murine models of COPD (Qi et al., 2023). These findings suggest that the occurrence of COPD is related to the expression of Cu-regulatory genes. CDKN2A is one of the genes involved in lung aging and is a Cu death regulator with the most frequent occurrence of somatic mutations (Wu et al., 2023). Therefore, targeting genes related to Cu metabolism may be an effective way to treat COPD. However, how these genes are regulated and whether abnormal interactions between them contribute to the development of COPD remain to be further explored.

### Asthma

Both Cu and Zn are important components in the development of certain allergic diseases, such as asthma, allergic rhinitis, and food allergies (Podlecka et al., 2022). Allergic asthma is a chronic lung disease characterized by reversible airway obstruction and inflammation primarily mediated by a type 2 immune response. Most studies suggest that Cu levels are elevated in patients with asthma (Di Toro et al., 1987; Gray et al., 2010). Studies have also shown that Cu and Zn levels are associated with the risk of developing asthma and can be used as routine biomarkers for asthma risk in patients with recurrent asthma (Uysalol et al., 2014). Short-term exposure to PM_2.5_-bound Cu in asthmatics is associated with a rapid decline in lung function in maximal mid-expiratory flow (MMEF) and a significant increase in IL-23 in the epithelial lining fluid (ELF), which, in addition to acting as a pro-inflammatory factor, promotes Th17 differentiation, disrupts the Th17/Treg balance, and increases IL-17 levels, leading to the development of asthma (Liu et al., 2023). Mice sensitized by intraperitoneal injection of ovalbumin and intranasally infused with CuO NPs exacerbated the development of asthma by increasing AHR, inflammatory cell count in bronchoalveolar lavage (BAL) fluid, mucus secretion, serum immunoglobulin E (IgE) levels, allergic inflammatory markers, including IL-5 and IL-13, and ROS production in BAL fluid (Park et al., 2016). Studies have shown that after exposure to CuO NPs in healthy mice, Th1 and Th2 cells are significantly reduced, T-bet + Treg cells are significantly increased, the Th2 immune response may still be reduced, and the TH1 response is suppressed 40 days after the end of the exposure period. Asthmatic mice show a Th2-dominated immune response and pathological asthma features, such as increased eosinophilic levels, mucus production, and perivascular infiltration. Asthmatic mice treated with immunotherapy, in contrast, showed a Th1-dominated immune response and reduced Th2 cell levels (Areecheewakul et al., 2023). The immunomodulatory effect of inhalation exposure to CuO NPs depends on the pre-exposure immune conditions. Therefore, the study of Cu in asthma should also consider the experimental model and basic immune status of patients.

Increased CP levels have been observed in patients with asthma (Winter et al., 2021). Another important Cu-containing enzyme is CuZn-SOD. CuZn-SOD activity is reduced in patients with asthma and allergic rhinitis (Sagdic et al., 2011). CuZn-SOD is located within the ciliated epithelium; therefore, any disturbance in Cu levels and CuZn-SOD may lead to further progression of oxidative stress (Kinnula and Crapo, 2003; Zajac, 2021). Individuals with asthma have higher levels of NO and nitrated products than healthy individuals, which is associated with disease severity. The nonenzymatic decomposition of S-nitrosothiols (RNSO) is mainly catalyzed by Cu^2+^ ions to produce NO and its corresponding disulfide. This suggests that elevated Cu levels in asthma may accelerate the consumption of RSNO, accompanied by an increase in NO levels. Therefore, RSNO metabolism, particularly Cu-mediated catalytic breakdown, may be a potential therapeutic target for asthma (Yates, 2001). Airway smooth muscle contractions are a major factor in the occurrence and development of asthma. Afrin *et al.* found in a study of rats that exposure to high free Cu^2+^ leads to acetylcholine (Ach)-induced excessive contraction of the tracheal ring. This contraction is caused by increased intracellular calcium contraction caused by activation of the voltage-operated Ca^2+^ channel, store-operated Ca^2+^ channel, and transient receptor potential channel (Afrin et al., 2022). However, the mechanism of Cu ions in asthma requires further investigation.

### PAH

PAH is a pulmonary vascular remodeling disease that leads to right ventricular failure and death within a few years of diagnosis. Patients with severe and progressive forms of PAH frequently present with complex angioobliterative vascular lesions that are refractory to vasodilator treatment.

As early as the 1980s, researchers identified elevated serum Cu as a possible cause or marker of PAH, and intravenous infusion of CuSO_4_ significantly increased pulmonary vascular resistance, indicating an important role of Cu in PAH formation (Ahmed and Sackner, 1985). Increased expression of the Cu-related genes *CTR1*, *ATP7A*, and *LOX* was observed in hypoxia-induced PAH mice. Increased CTR1 expression has been observed in macrophages in response to hypoxia (White et al., 2009b). Bogaard et al. (2012) showed that after exposure to the VEGF receptor tyrosine kinase inhibitor SU5416 and hypoxia (SuHx model), rats developed endothelial cell proliferation and severe pulmonary hypertension only when provided with adequate amounts of Cu in their diet. Chronic TM therapy reversed pulmonary hypertension and vascular occlusion in SuHx rats, and its therapeutic effect was associated with reduced survivin expression, increased production of the proapoptotic sphingoid metabolite ceramide, and increased expression of apoptosis-inducing factor and Bim in B-cell leukemia/lymphoma protein family members. TM also inhibits the proliferation of human lung endothelial cells cultured from patients with PAH and controls. Consistent with the idea that Cu is required for the formation of vasoproliferative lesions, stopping its restriction led to the recurrence of lesions and PAH, whereas Cu-chelating agents slowed the growth of lung endothelial cells (including cells cultured from patients with idiopathic PAH) (Bogaard et al., 2012). Wang et al. detected that the expression of the Cu metabolism-related genes, DNA damage-inducible transcript 3 (DDIT3), NFKB inhibitor alpha (NFKBIA), oncostatin M (OSM), and prostaglandin E receptor 4 (PTGER4), was downregulated in PAH, and a model based on the four genes could potentially be a biomarker for PAH (Wang et al., 2023). Cu may lead to increased expression of DDIT3, which promotes monocrotaline (MCT) vascular remodeling in PH (Jiang et al., 2021). However, DDIT3 was upregulated in a validated cohort of patients with PAH, possibly because of sample heterogeneity or limited sample size. Therefore, additional data were required for validation. NFKBIA inhibits NF-κB (Sogkas et al., 2020), epithelial-mesenchymal transition (EMT), cell migration, proliferation, and invasion. NF-κB plays an important role in PAH and can be regulated by Cu (Price et al., 2013; Kenneth et al., 2014), and inhibition of NF-κB prevents MCT-induced PAH in mice (Li et al., 2014). Using a rat model of hypoxic pulmonary hypertension (HPH) and primary pulmonary microvascular endothelial cells (PMVECs), Wang et al. demonstrated that phosphoinositide-dependent protein kinase 1 (PDK1)-induced PMVECs proliferation inhibits apoptosis by participating in pulmonary vascular remodeling, ultimately leading to HPH by regulating PTEN-induced kinase 1 (PINK1)-mediated mitochondrial autophagy signal transduction. Thus, PINK1 may be a novel therapeutic target for HPH control (Wang et al., 2023). Cuproptosis is related to TCA, and the abnormal TCA cycle plays a role in the development of PAH. Therefore, the role of cuproptosis in PHA should be further studied and analyzed.

### Pulmonary fibrosis

IPF is a progressive interstitial lung disease. The wide heterogeneity of clinical presentations and symptoms leads to a high variability in the course and response to treatment. Without treatment, the average life expectancy of patients with IPF is 3–5 years after diagnosis (Raghu et al., 2018). The regulatory role of Cu ions in lung fibrosis has been previously demonstrated. Cu has been reported to be elevated in the BAL of patients with pulmonary fibrosis (Bargagli et al., 2017). In addition, the expression of LOXL2 was increased in fibrotic lungs, and a LOXL2 inhibitory antibody protected against bleomycin-induced lung injury (Barry-Hamilton et al., 2010). Elevated urinary Cu levels were recently reported to be directly correlated with a higher risk of fibrotic changes in the lungs (Chiou et al., 2023). scRNA-seq data from animal models of pulmonary fibrosis and human samples suggested that CRGs are maintained at a certain level in healthy controls. However, the expression levels of CRGs gradually decreased as the fibroblasts differentiated from a quiescent state into myofibroblasts. In addition, cell differentiation was accompanied by metabolic reprogramming, with a gradual decrease in the TCA cycle and lipid metabolism and a gradual increase in mitochondrial metabolism. Studies have demonstrated that cuproptosis levels are negatively correlated with fibrosis levels in both animals and humans, suggesting a potential role in pulmonary fibrosis (Li et al., 2022). In experimental models, inhalation of Cu nanoparticles in mice caused pulmonary toxicity and pulmonary fibrosis (Lai et al., 2018), and macrophages of patients exposed to asbestos had high levels of Cu and Zn-SOD/SOD1 and produced high levels of H_2_O_2_, whereas Cu, Zn-SOD/SOD1 deficient mice were protected from asbestos-induced lung damage and pulmonary fibrosis after exposure to asbestos in the trachea (He et al., 2011; He et al., 2016). Individuals exposed to CuSO_4_ have a higher risk of granulomatous lung disease and progressive mass fibrosis (Skoczyńska et al., 2016).

Airway epithelial cells transform into mesenchymal-type cells under stimulation, known as EMT, which is a key stage in the development of fibrosis. EMT is a reversible process, and Cu signaling is essential for EMT induction. Guo *et al.* demonstrated that intragastric exposure to CuSO_4_ at 10–40 mg/kg for 3 weeks induced EMT and fibrotic changes in mouse lungs (Guo et al., 2021). Gaun *et al.* recently reported that drinking water for 16 weeks in mice exposed to 250 ppm CuSO_4_ resulted in lung damage, including altered alveolar thickness and collagen deposition (Gaun et al., 2023). In addition, intranasal administration of CuO NP promoted the accumulation of collagen in lung tissue and the expression ofα-smooth muscle actin (α-SMA), a marker of progressive fibrosis, and induced pulmonary fibrosis changes at 28 days after administration (Lai et al., 2018). *In vitro*, 100 μM CuSO_4_ treatment of lung epithelial cells consistently reduced cell viability of alveolar type (A549) and human bronchial epithelial cells (HBE). CuSO_4_ promotes EMT, induces autophagy, increases LC3 and PINK expression, and decreases p62 expression. N-acetylcysteine inhibition of ROS reversed CusO_4_-induced PINK1, LC3, and Snail expression. Inhibition of autophagy by chloroquine reverses CuSO_4_-induced EMT changes. Natural flavonoids, particularly kaempferol and fustin, inhibit Cu-induced EMT. These results suggest that Cu is a risk factor for pulmonary fibrosis that activates the ROS-autophagy pathway and that flavonoids can reverse this process (Chiou et al., 2023). In conclusion, these studies provide *in vitro* and *in vivo* evidence of a causal relationship between Cu exposure and pulmonary fibrosis and suggest that EMT changes are a key mechanistic event in Cu-induced pulmonary fibrosis. The EMT is a therapeutic target for fibrotic diseases (Di Gregorio et al., 2020).

There is growing evidence that proteases and anti-proteases are involved in IPF pathogenesis (Craig et al., 2015). Collagen overexpression and deposition are important pathological features of IPF (Craig et al., 2015). Elevated intracellular Cu ions activate LOX to enhance the crosslinking of collagen and elastin (Janssen et al., 2018), and LOX activity is increased in fibrotic lung diseases, such as IPF (Tjin et al., 2017). Several fibrosis markers (COL1A1, MMP7, LOXL2, and N-cadherin) were upregulated in the lung epithelial cells after Cu treatment. LOXL2 is a Cu-dependent amine oxidase that plays a critical role in matrix remodeling and fibrosis (Chiou et al., 2023). Expression levels of LOXL2 are highly correlated with the severity of pulmonary fibrosis, and LOXL2 expression is significantly increased in serum, lung homogenate, and lung tissue of bleomycin-induced pulmonary fibrosis mice, critical for the transformation of fibroblasts into myofibroblasts via the TGF-β/Smad pathway. Blocking LOXL2 significantly attenuates fibrosis in bleomycin-induced mouse fibrosis models (Wen et al., 2018). In human studies, serum LOXL2 expression is associated with the risk of IPF disease progression and death (Chien et al., 2014). This suggests that LOXL2 is not only a marker of fibrosis but is also critical for mediating Cu-induced fibrosis. Intracellular LOX promotes AP-1 induction and transcriptional regulation of target genes such as collagen, fibronectin, and IL-6. LOX has been shown to alter lung fibroblast transcriptomes (Mižíková et al., 2017). LOX levels and activity can be used as novel biomarkers for fibroproliferative diseases and to monitor responses to therapy. Therefore, it is hypothesized that LOX is a new therapeutic target for fibroproliferative diseases and that the development of therapies to reduce LOX levels and activity may improve organ fibers (Nguyen et al., 2021). In the lung tissue of patients with IPF, the elastin fiber content increases, which is correlated with the degree of fibrotic lesions and collagen content in the lung tissues. In addition, elastin levels correlated negatively with exertional spirometry and positively with reduced lung function in patients with IPF (Enomoto et al., 2013). This may be attributed to the elastin repair mechanism in patients with IPF. Among the multiple elastin repair steps, the synthesis of the cupric-dependent enzyme LOX and crosslinking of the pro-elastin polymer appear to be the most important. Janssen et al. speculated that the difference between pulmonary fibrosis and emphysema depends on the local availability of Cu to activate sufficient LOX for elastin crosslinking (Janssen et al., 2018). Whether there is a theoretical basis for treating emphysema using inhaled Cu needs to be explored.

### Pulmonary infections

Cu is an essential micronutrient for both pathogens and infected animal hosts. In addition to being essential for the function of the immune system in humans, copper neutralizes infectious DNA and RNA viruses and activates autophagy (Zischka and Kroemer, 2020; Szewc et al., 2022). During infection, invading bacterial pathogens must obtain essential metals such as Zn, manganese, Fe, and Cu from their hosts to colonize and replicate. Animal hosts can also develop nutritional immunity by limiting Cu nutrients to prevent pathogen growth (Besold et al., 2016).

Cu levels are elevated at sites of pulmonary infection (Besold et al., 2016). For example, *Mycobacterium tuberculosis* infection produces granulomas in the lungs of animals and Cu is significantly higher than that in the surrounding tissues (Ward et al., 2010). The Cu detoxification mechanisms of *Mycobacterium tuberculosis* are critical for pulmonary pathogenesis (Wolschendorf et al., 2011). In addition, the Cu-responsive RicR regulon contributes to *M*. *tuberculosis* virulence (Shi et al., 2014). *Cryptococcus* neoformans induces the expression of two metallothionein genes, Cu detoxification machinery chromomethylase (CMT) 1 and CMT2, under high Cu conditions (Ding et al., 2013). One possible source of pulmonary Cu is macrophages. The mechanism of increased Cu may involve increased Cu uptake by macrophage CTR1 Cu permease and the activation of the Cu ATPase ATP7A in phagolysosomes (Ding et al., 2013). Macrophages can attack invading microorganisms with high Cu levels and are dense in the lungs at sites of infection with coronavirus disease 2019 (COVID-19) and *S. pneumoniae* (Knoll et al., 2021). In a recent experiment by Johnson *et al.*, Cu toxicity stress in the lungs of *Streptococcus pneumoniae* improved after the depletion of pulmonary macrophages, strongly suggesting that macrophages are associated with this enhanced Cu response. In contrast, lung bacterial colonization is greatly reduced in *S*. *pneumoniae* mutants that cannot export Cu via CopA Cu-transporting ATPase (Johnson et al., 2015).

However, Cu can also be toxic to cells because of its redox properties and ability to disrupt the active sites of metalloproteins such as Fe-S enzymes. Owing to these toxicities, Cu is an effective antimicrobial agent, as evidenced by significant differences in serum Fe, Zn, Cu, and Se concentrations in patients with tuberculosis (TB) during infection and anti-TB treatment compared to that of the controls (Sepehri et al., 2018). Cu homeostasis is also required for the virulence of the fungal pathogen *C. neoformans*. The host may attack *C. neoformans* with elevated Cu levels in the lungs via the pulmonary macrophages. In contrast, *C. neoformans,* as previously stated, responds to elevated pulmonary Cu concentrations during infection by strongly inducing the expression of two metallothionein genes, CMT1 and CMT2, in a time-dependent manner (Besold et al., 2016). Therefore, *C. neoformans* mutants lacking MT show poor pulmonary infectivity (Ding et al., 2013; García-Santamarina and Thiele, 2015). Recent studies have shown that microplusin, a Cu-chelating antimicrobial peptide, is a potential therapeutic agent against *C. neoformans* (Waterman et al., 2012). Cu homeostasis plays a crucial role in *A. fumigatus* virulence. Cu and ROS in macrophages are closely related to the killing of *Aspergillus fumigatus*, which has evolved complex mechanisms to regulate Cu homeostasis (Song et al., 2019).

**FIGURE 4:**
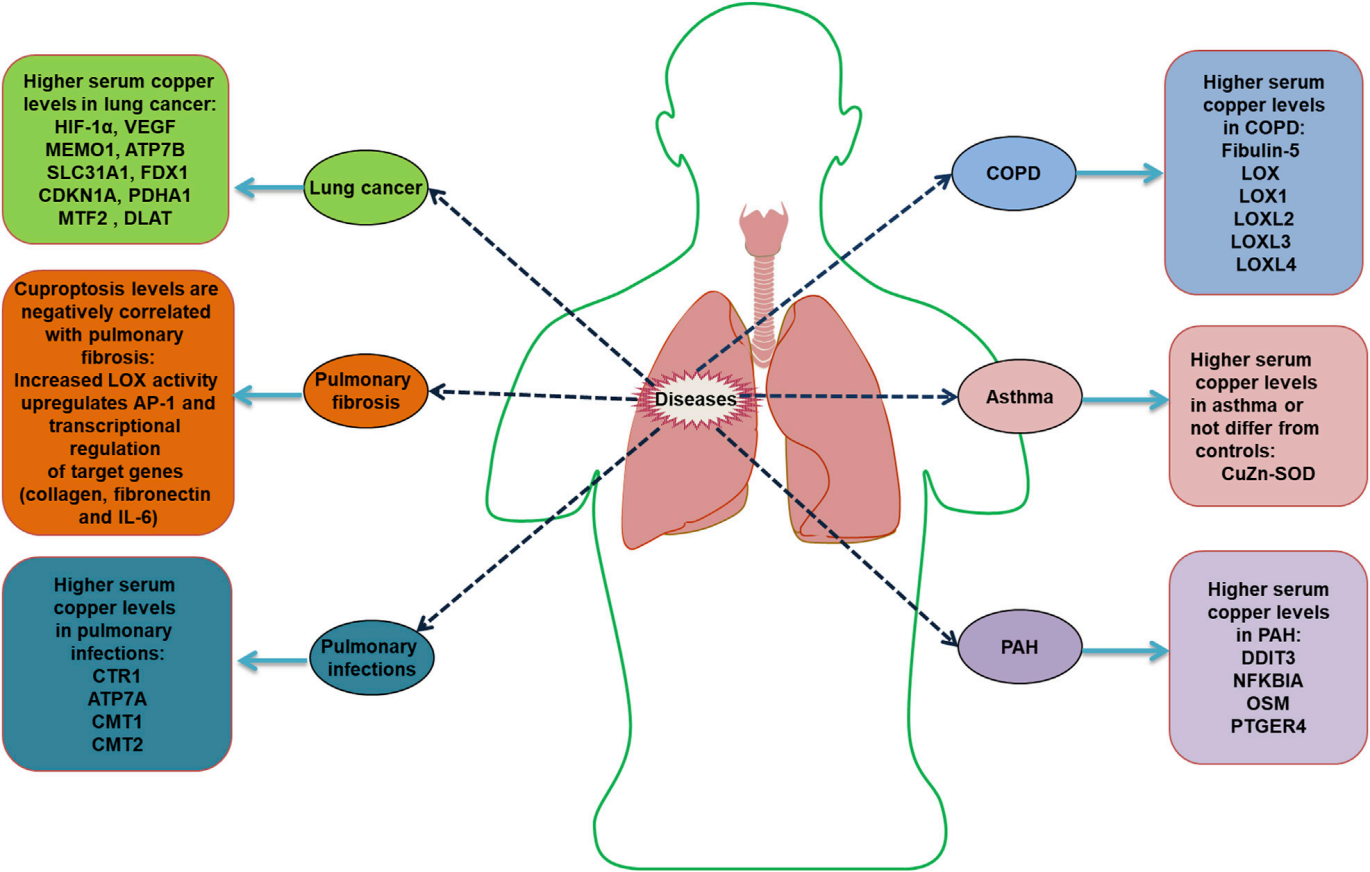
Dysregulation of copper homeostasis in respiratory diseases HIF-1α, hypoxia-inducible factor 1α; VEGF, vascular endothelial growth factor; MEMO1, mediator of cell motility 1; ATP7B, ATPase copper transporting beta; SLC31A1, solute carrier family 31 member 1; FDX1, ferredoxin 1; CDKN1A, cyclin dependent kinase inhibitor 1A; PDHA1, pyruvate dehydrogenase E1 subunit alpha 1; MTF2, metal response element binding transcription factor 2; DLAT, dihydrolipoamide S-acetyltransferase; LOX, lysyl oxidase; IL, interleukin; CTR1, Cu transport protein 1; ATP7A, ATPase copper transporter 7A; CMT, chromomethylase; LOXL, lysyl oxidase like; CuZn-SOD, copper-zinc superoxide dismutase; DDIT3, DNA damage-inducible transcript 3; NFKBIA, NFKB inhibitor alpha; OSM, oncostatin M; PTGER4, prostaglandin E receptor 4.

Cu levels are valuable for the prognosis of patients with COVID-19. An analysis of whole-blood trace elements in patients with COVID-19 in Wuhan showed that serum Cu levels generally increased in patients with severe disease (Zeng et al., 2021). Increased serum Cu^2+^ and decreased Zn^2+^ levels in patients with COVID-19 at an early stage were mainly associated with inflammatory responses (Ivanova et al., 2022). Hackler et al. found that serum Cu and Se levels in patients with COVID-19 contribute to the prediction of patient prognosis. Supplementation with Cu-containing adjuvants in patients diagnosed with Cu or Se deficiency may positively affect disease regression (Hackler et al., 2021). However, studies have also not indicated that Zn, Se, Cu, and vitamin K supplementation can prevent SARS-CoV-2 infection, critical illness, or COVID-19 hospitalization (Sobczyk and Gaunt, 2022). Cu has antiviral properties at two levels: enhancing immune system components to fight infection and direct exposure to the virus (van Doremalen et al., 2020). Poor Cu intake (even if severe deficiency is not reached) is associated with reduced T cell proliferation and abnormal macrophage phagocytosis (Percival, 1998). Galmés et al. also summarized the contribution of Cu as a nutritional and immune-enhancing factor in the context of the COVID-19 pandemic (Galmés et al., 2020). However, an excessive intake of this mineral can negatively affect the immune system (Liu et al., 2022). *In vitro* studies have shown that Cu ions block essential proteins for SARS-CoV-1 replication (Báez-Santos et al., 2015). Further clinical studies and the specific effects of Cu on patients with consideration of their individual characteristics and interactions with other minerals and nutrients, are needed to specifically distinguish whether Cu is a single factor causing changes in the results. Cu is a candidate for future studies of its therapeutic efficacy in combination with other drugs, such as N-acetylcysteine, colchicine, or raltegravir, as a treatment strategy for COVID-19 (Andreou et al., 2020). Cu homeostasis components related to respiratory diseases are listed in Table 2. The dysregulation of Cu homeostasis in respiratory diseases is summarized in Figure 4.

**TABLE 2:** Cu homeostasis components related to respiratory diseases.

Diseases		Clinical values	References
Lung cancer	Human studies	Serum copper content increased; Cu/Zn contributes to the assessment of prognosis and disease severity in patients with lung cancer	Wang et al. (2021)
COPD	Human studies	The higher the serum copper level in the general population, the lower the FEV1	Pearson et al. (2005)
		Elevated serum Cu/Zn ratios and Cu concentrations are associated with an increased risk of COPD in men	Fei et al. (2022)
		COPD smokers and non-smokers: Differences in Cu, MDA and serum PON1 activity	Sarioglu et al. (2020)
		AECOPD patients and smokers: Cu and Zn levels increased in COPD patients with acute recombination, and Cu/Zn ratio decreased significantly. Lipid hydroperoxide, malondialdehyde; Cu and Zn levels were negatively correlated with FEV1 and FVC levels	Bozkus et al. (2016)
		Copper levels in EBC were lower in stable COPD patients than in healthy non-smokers	Mutti et al. (2006)
	Animal studies	Airway epithelial cells of COPD rats/COPD patients: increased copper content; Enrichment of genes related to copper metabolism	Qi et al. (2023)
		Male Sprague-Dawley rats: Copper deficiency induced emphysematous changes; HIF-1α activity was decreased and VEGF expression was downregulated	Mizuno et al. (2012)
Asthma	Human studies	PM_2.5_-bound Cu is associated with a rapid decline in lung function in MMEF and a significant increase in IL-23 in ELF	Liu et al. (2023)
		Serum copper content increased	Wu et al. (2023)
		Cu, Zn-SOD decreased	Comhair et al. (2005)
		CP increase	Winter et al. (2021)
	Animal studies	After intraperitoneal ovalbumin sensitization, CuO NPs infusion increased the number of inflammatory cells in AHR, BAL fluid, mucus secretion, serum IgE level, and IL-5, IL-13 and ROS in BAL fluid	Park et al. (2016)
		In mice: Th1 and Th2 cells were significantly reduced after exposure to CuONPs. Asthmatic mice showed a Th2-led immune response, increased eosinophils levels, mucus production, and perivascular infiltration	Areecheewakul et al. (2023)
		In rats: Exposure to high free Cu^2+^ results in ACh-induced hyperconstriction of the tracheal ring	Afrin et al. (2022)
PAH	Human studies	The mean serum copper level was significantly increased in patients with pulmonary hypertension	Ahmed and Sackner (1985)
	Animal studies	Hypoxic-induced PAH mice: increased expression of copper-related genes, CTR1, copper efflux pump ATP7A and lysyl oxidase	White et al. (2009b)
		SuHx model rat/human lung endothelial cells: endothelial cell proliferation and severe pulmonary hypertension occurred only when copper was sufficient; Pulmonary hypertension and vascular occlusion in SuHx rats were reversed after chronic TM treatment	Bogaard et al. (2012)
Pulmonary fibrosis	Human studies	The copper in BAL liquid is elevated	Bargagli et al. (2017)
		LOXL2 expression increased	Barry-Hamilton et al. (2010)
	Animal studies	Mice: Cu, Zn-SOD/SOD1 deficient mice are protected from asbestos-induced lung damage	He et al. (2011), He et al. (2016)
		In mice: Bleomycin increased LOXL2 expression	Aumiller et al. (2017)
		Mice: LOXL2 inhibitory antibody protects against bleomycin-induced lung injury	Barry-Hamilton et al. (2010)
		mice: exposure of CuSO4 at 10–40 mg/kg intragastrically for 3 weeks induced EMT and fibrotic changes in mice lungs	Guo et al. (2021)
		C57BL/6 mice: Copper oxide nanoparticles can induce ROS increase, epithelial cell apoptosis, and promote the accumulation of collagen in lung tissue and the expression of α-SMA, a marker of progressive fibrosis	Lai et al. (2018)
		Alveolar type (A549) and HBE: EMT induced autophagy, LC3, PINK expression increased, p62 expression decreased	Chiou et al. (2023)
		Bleomycin-induced pulmonary fibrosis mouse model: During the differentiation of resting fibroblasts into myofibroblasts, the genes associated with pulmonary fibrosis are progressively downregulated	Li et al. (2022b)
Pulmonary infection	Human studies	Copper reactive transcription factors control genes that are important for *mycobacterium tuberculosis* virulence	Ward et al. (2010)
		Serum copper is elevated during infection	Wolschendorf et al. (2011)
	Animal studies	Mice: Copper-responsive RicR regulon contributes to *mycobacterium tuberculosis* virulence	Shi et al. (2014)
		C. neoformans induced the expression of two metallothionein genes CMT1 and CMT2 under Cu condition	Ding et al. (2013)

FEV1, forced expiratory volume in 1s; FVC, forced vital capacity; MDA, malonaldehyde; PON1, paraoxonase 1; COPD, chronic obstructive pulmonary disease; EBC, exhaled breath condensate; HIF-1α, hypoxia-inducible factor 1α; VEGF, vascular endothelial growth factor; MMEF, maximal mid-expiratory flow; IL, interleukin; ELF, epithelial lining fluid; NPs, nanoparticles; IgE, immunoglobulin E; ROS, reactive oxygen species; AHR, anti-hydroxy radical; BAL, bronchoalveolar lavage; ACh, acetylcholine; PAH, pulmonary arterial hypertension; CTR1, copper uptake transporter; ATP7A, ATPase, copper transporter 7A; TM, tetrathiomolybdate; LOXL2, lysyl oxidase like 2; EMT, epithelial-mesenchymal transition; α-SMA, α-smooth muscle actin; HBE, human bronchial epithelial cells; PINK, PTEN, induced kinase 1; CMT, cu detoxification machinery.

## Potential for Cu-targeted therapeutic approaches

Cu metabolism is important in various processes such as inflammation, oxidative stress, and immunity. As dysregulation of Cu homeostasis is associated with many lung diseases, treatment to correct systemic and/or local Cu levels may be effective in treating these diseases. Lee et al. found that the mortality rate was 5.6% in critically ill patients with increased serum Cu concentrations and 50.0% in patients with decreased serum Cu concentrations after supplementation with standard trace elements for 2 weeks in critically ill patients (Lee et al., 2019).

In addition to direct Cu supplementation, the modification of Cu homeostasis has become a strategy for combating tumors. There are currently two main mechanisms of action. One is the use of Cu chelators to reduce Cu ion levels and inhibit tumor cell proliferation (Tsang et al., 2020; Cui et al., 2021). The other is the transfer of Cu ions from the extracellular to the intracellular level via their carriers, which increases intracellular Cu^2+^ levels and induces oxidative stress, leading to tumor cell death (Li, 2020; ODay et al., 2009; ODay et al., 2013). Therefore, the use of Cu-induced cell death as a target for Cu-based therapy is expected to be a new approach to cancer treatment.

The development of new agents that trigger Cu death, such as the GOx@[Cu(tz)] nanomaterials constructed by Xu et al., is important for designing Cu-death-based therapeutic strategies for tumors. When specifically released into tumor cells, GOx catalyzes the oxidation of glucose in tumor cells and blocks the energy supply to tumor cells. Simultaneous depletion of GSH and glucose leads to increased susceptibility of tumor cells to GOx@[Cu(tz)]-mediated cuproptosis, resulting in significant oligomerization of DLAT proteins. In contrast, the addition of cuproptosis inhibitors and Cu ion chelators restores Cu-induced cell death (Xu et al., 2022).

Mitochondrial dysfunction has been implicated in the pathogenesis of lung diseases such as COPD, IPF, asthma, and lung cancer. Cu is essential for normal mitochondrial function. Chiou et al. demonstrated that Cu induces mitochondrial autophagy in lung epithelial cells, and the inhibition of autophagy blocks Cu-induced EMT (Chiou et al., 2023). Non-toxic Cu overload stimulates mitochondrial renewal through mitochondrial autophagy and biogenesis (Ruiz et al., 2016). Some studies have demonstrated that metformin targets mitochondrial Cu, induces ROS production, causes mitochondrial dysfunction and apoptosis, and blocks EMT (Müller et al., 2018). Cuproptosis is primarily induced by mitochondrial Cu. Therefore, mitochondrial Cu chelators may be interesting therapeutic targets. Cui et al. developed a safe mitochondria-targeted copper-depleting nanoparticle (CDN) and tested it against triple-negative breast cancer (TNBC). In mouse models, mitochondrial dysfunction induced by CDN administration and energy and nutrient deficiencies caused by Cu consumption, elevated oxidative stress, and mitochondrial membrane rupture contribute to the apoptosis of TNBC cells, inhibit tumor growth, and significantly improve the survival rate. Meanwhile, CDN administration is beneficial for the deprivation of Cu in cancer cell mitochondria, rather than systemic depletion; therefore, it has low toxicity (Cui et al., 2021).

Targeting Cu ions also has important applications in the treatment of non-neoplastic respiratory diseases. Chelating Cu with TM and the knockdown of CTR have also been effective in ameliorating bleomycin-induced pulmonary fibrosis, providing options for patients with IPF who do not respond to other therapies (Brewer et al., 2003). For example, in a mouse model of bleomycin-induced IPF, the administration of TM induced a reduction in serum Cu cyanobacteria, leading to a corresponding reduction in pulmonary fibrosis (Brewer et al., 2003). TM benefits IPF by reducing collagen I expression and accumulation and acting on Cu-dependent LOX expression (Ovet and Oztay, 2014; Baldari et al., 2020). In a clinical study on breast cancer, 51 patients treated with oral TM for 2 years showed decreased LOX activity against collagen crosslinking and lung metastasis (Niu et al., 2020). d-Penicillamine is a potent chelator of Cu (Lipsky, 1984) and its application reduces skin involvement in patients with diffuse cutaneous systemic sclerosis and improves kidney, heart, and lung involvement (Steen et al., 1982).

In addition to treatment, mouse models of various cancers have been used as diagnostic tools to investigate ^64^Cu (Denoyer et al., 2015). Preliminary results confirmed that ^64^Cu was selectively concentrated in malignant tissues rather than in inflammatory tissues, suggesting a potential role for this radionuclide as a specific tumor marker. However, its therapeutic role remains unknown (Capriotti et al., 2022). In humans, García-Pérez et al. observed high ^64^Cu uptake in large peripheral primary lung cancer lesions on positron emission tomography (PET)/computerized tomography (CT) images, comparable to β-2-[^18^F]-fluoro-2-deoxy-D-glucose (FDG) uptake. High uptake of ^64^Cu was detected in 36% of primary tumors and 27% of lymph node metastases. It is suggested that ^64^Cu^2+^ ions can be used as valid biomarkers for the noninvasive assessment of cancer using PET imaging. Furthermore, tumor uptake of ^64^Cu and mouse CTR1 levels are correlated (Chakravarty et al., 2016). Therefore, ^64^Cu-PET/CT could be used to avoid ineffective platinum-based chemotherapy in patients with non-^64^Cu-avid lung cancer lesions (Léger et al., 2022).

However, the mechanisms underlying Cu metabolism and Cu-induced cell death remain unclear. Therefore, further studies on the mechanisms of Cu metabolism homeostasis and related signaling pathways are of great significance for the diagnosis and treatment of respiratory diseases. Cu chelators are expected to become an important component of adjuvant therapy for various diseases.

## Conclusions and prospects

In summary, several lung diseases, particularly lung cancer, are associated with altered Cu levels and/or lung homeostasis. Cu homeostasis in the body is maintained through the regulation of Cu uptake, transport, and excretion. Cuproptosis is a new form of regulated cell death that is mainly associated with disorders of TCA cycle metabolism. This form of death is not completely independent of other regulated forms however may be closely linked to them. This review highlights the need to further investigate the precise mechanisms of Cu interactions with infections, immune inflammation, etc., in the context of respiratory diseases, and to explore the potential of therapeutic strategies for Cu, cuproptosis, and other related effects. Many respiratory diseases are comorbid. For example, COPD and interstitial lung fibrosis are often associated with tumors. Therefore, whether cuproptosis is a crossover point in their pathogenesis or a crossover target for treatment requires further experimentation and mechanistic exploration. The discovery of cuproptosis contributes to a deeper understanding of Cu metabolic diseases and their underlying molecular mechanisms. This in turn will be of value in improving the understanding of Cu metabolism and cuproptosis mechanisms, and in screening relevant drugs for treating Cu metabolic diseases. Further experimental and clinical studies are needed to target Cu therapy for respiratory diseases.
